# Supplementary material for: The mutational landscape of the SCAN‐B real‐world primary breast cancer transcriptome
Source: EMBO Mol Med. 2020 Sep 14;12(10):e12118. doi: 10.15252/emmm.202012118 (PMC7539222; doi:10.15252/emmm.202012118)
Supplement: Supplementary file 1 — Expanded View Figures PDF [file EMMM-12-e12118-s001.pdf]

# Expanded View Figures

**Figure EV1. Overview of frequently mutated genes in targeted DNA-seq and RNA-seq across 275 ABiM samples.**

A, B Waterfall plot of the 20 most mutated genes (rows) across 275 ABiM samples (columns) in (A) targeted DNA-seq and (B) RNA-seq. Genes are ranked by variant frequency. Samples are sorted by histological subtype and alteration occurrence. Mutations are colored by predicted functional impact.


© The Authors

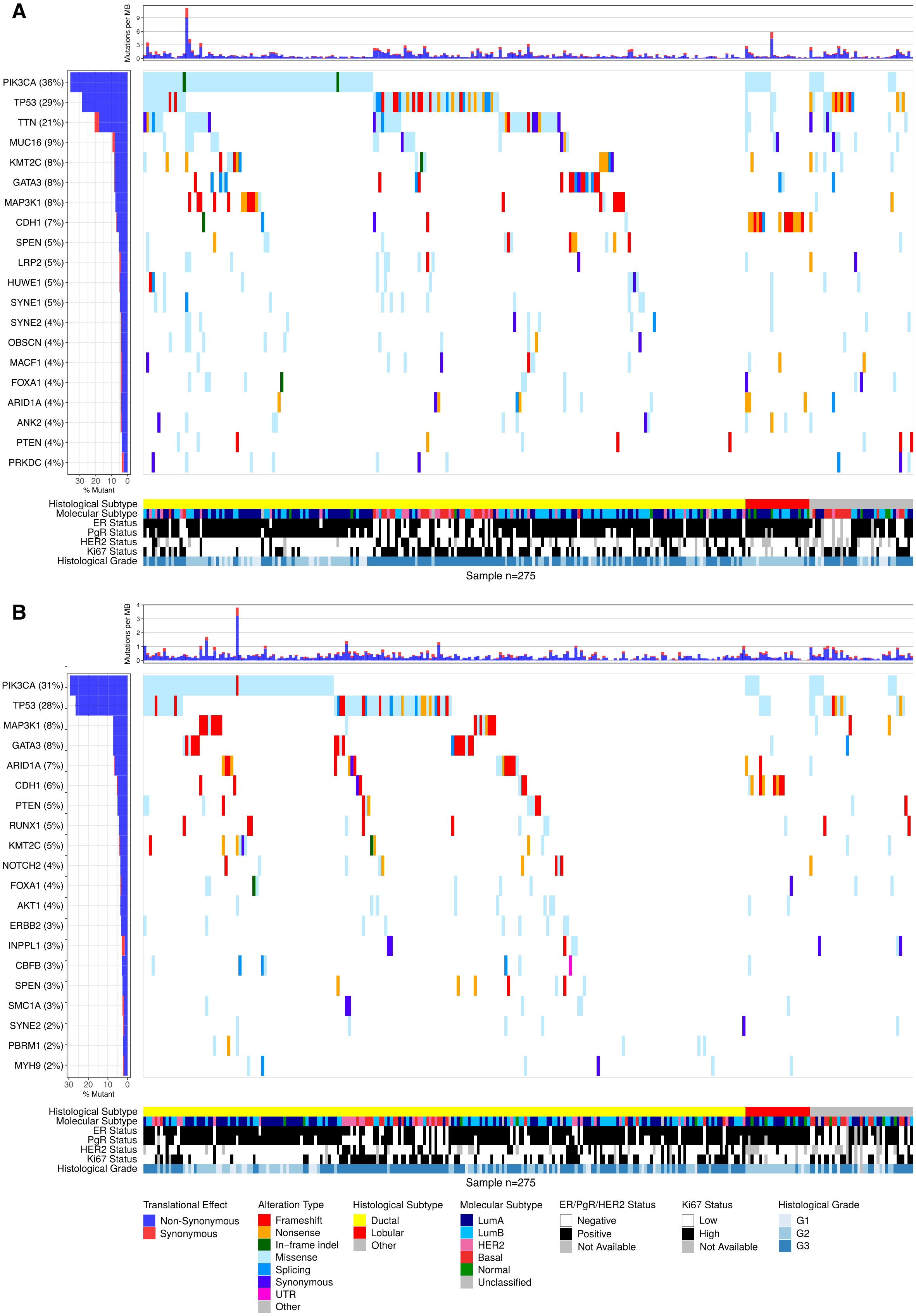
A
Mutations per MB
PIK3CA (36%)
TP53 (29%)
TTN (21%)
MUC16 (9%)
KMT2C (8%)
GATA3 (8%)
MAP3K1 (8%)
CDH1 (7%)
SPEN (5%)
LRP2 (5%)
HUWE1 (5%)
SYNE1 (5%)
SYNE2 (4%)
OBSCN (4%)
MACF1 (4%)
FOXA1 (4%)
ARID1A (4%)
ANK2 (4%)
PTEN (4%)
PRKDC (4%)
% Mutant
Histological Subtype
Molecular Subtype
ER Status
PgR Status
HER2 Status
Ki67 Status
Histological Grade
Sample n=275
B
Mutations per MB
PIK3CA (31%)
TP53 (28%)
MAP3K1 (8%)
GATA3 (8%)
ARID1A (7%)
CDH1 (6%)
PTEN (5%)
RUNX1 (5%)
KMT2C (5%)
NOTCH2 (4%)
FOXA1 (4%)
AKT1 (4%)
ERBB2 (3%)
INPPL1 (3%)
CBFB (3%)
SPEN (3%)
SMC1A (3%)
SYNE2 (2%)
PBRM1 (2%)
MYH9 (2%)
% Mutant
Histological Subtype
Molecular Subtype
ER Status
PgR Status
HER2 Status
Ki67 Status
Histological Grade
Sample n=275
Translational Effect
Non-Synonymous
Synonymous
Alteration Type
Frameshift
Nonsense
In-frame indel
Missense
Splicing
Synonymous
UTR
Other
Histological Subtype
Ductal
Lobular
Other
Molecular Subtype
LumA
LumB
HER2
Basal
Normal
Unclassified
ER/PgR/HER2 Status
Negative
Positive
Not Available
Ki67 Status
Low
High
Not Available
Histological Grade
G1
G2
G3

**Figure EV1.**

**Figure EV2. Impact of gene mutations on overall survival across clinical subgroups.**

A–E Overall survival (OS) of patients with tumors containing mutations in the genes (A) *TP53*, (B) *PIK3CA*, (C) *ERBB2*, and (D) *PTEN.* (E) OS by *PTEN*-MutExp genotype ("low" defined as *PTEN* mutation or *PTEN* expression in the lower quartile across the cohort, "normal" otherwise) stratified by the clinical patient subgroups $HoR^+/HER2^-$ ($HoR^+$ when $ER^+$ and $PgR^+$, $HoR^-$ otherwise; *n* = 2,134), $HoR^+/HER2^+$ (*n* = 230), $HoR^-/HER2^+$ (*n* = 104), and TNBC (*n* = 137). Specific treatments in these groups are detailed in Table EV4. In each Kaplan–Meier plot, wild-type (wt) and normal cases are plotted in blue, mutated (mut) and low cases are plotted in red, the log-rank *P* value is given, and the hazard ratio (HR) for mutation/low is given with a 95% CI and after univariable and multivariable (MV) Cox regression adjustment. Covariables included in the MV analysis were age at diagnosis, lymph node status, tumor size, and the variables denoted by the following symbols: ¤, ER, PgR, and NHG; #, NHG. ER, estrogen receptor; HER2, human epidermal growth factor receptor 2; HoR, hormone receptor; NHG, Nottingham histological grade; PgR, progesterone receptor; TNBC, triple-negative breast cancer.

 © The Authors

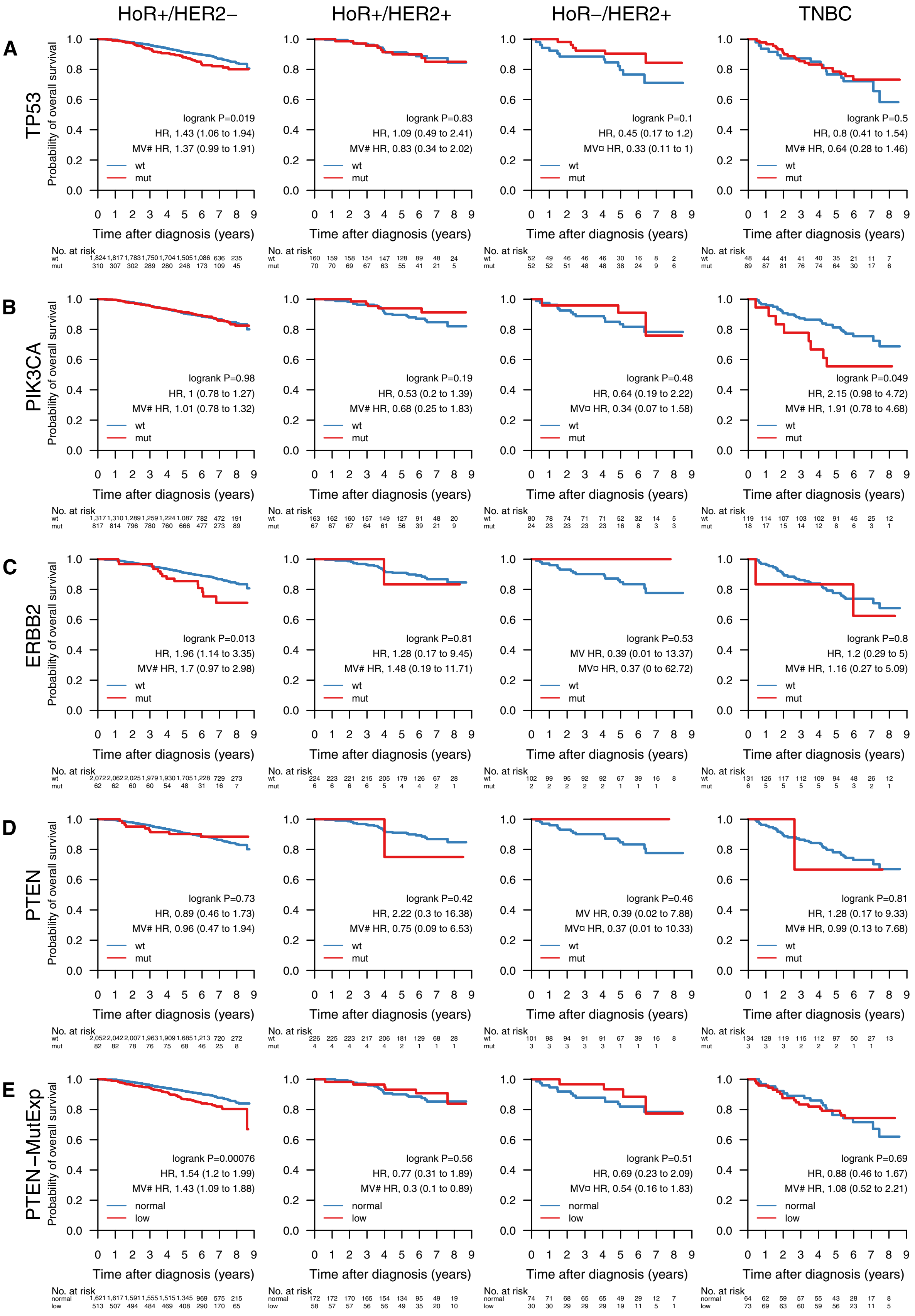

**Figure EV2.**

**Figure EV3. Impact of pathway mutations on overall survival across clinical subgroups.**

A–E Overall survival of patients with tumors containing mutations in pathways (A) WNT signaling, (B) Hedgehog signaling, (C) cell cycle, (D) p53 independent DNA damage repair, and (E) TGFβ signaling, stratified by the clinical patient subgroups $HoR^+/HER2^-$ ($HoR^+$ when $ER^+$ and $PgR^+$, $HoR^-$ otherwise; $n$ = 2,134), $HoR^+/HER2^+$ ($n$ = 230), $HoR^-/HER2^+$ ($n$ = 104), and TNBC ($n$ = 137). Specific treatments in these groups are detailed in Table EV4. In each Kaplan–Meier plot, wild-type (wt) cases are plotted in blue, mutated (mut) cases are plotted in red, the log-rank $P$ value is given, and the hazard ratio (HR) for mutation is given with a 95% CI and after univariable and multivariable (MV) Cox regression adjustment. Covariables included in the MV analysis were age at diagnosis, lymph node status, tumor size, and the variables denoted by the following symbols: ¤, ER, PgR, and NHG; #, NHG. ER, estrogen receptor; HER2, human epidermal growth factor receptor 2; NHG, Nottingham histological grade; PgR, progesterone receptor.


© The Authors

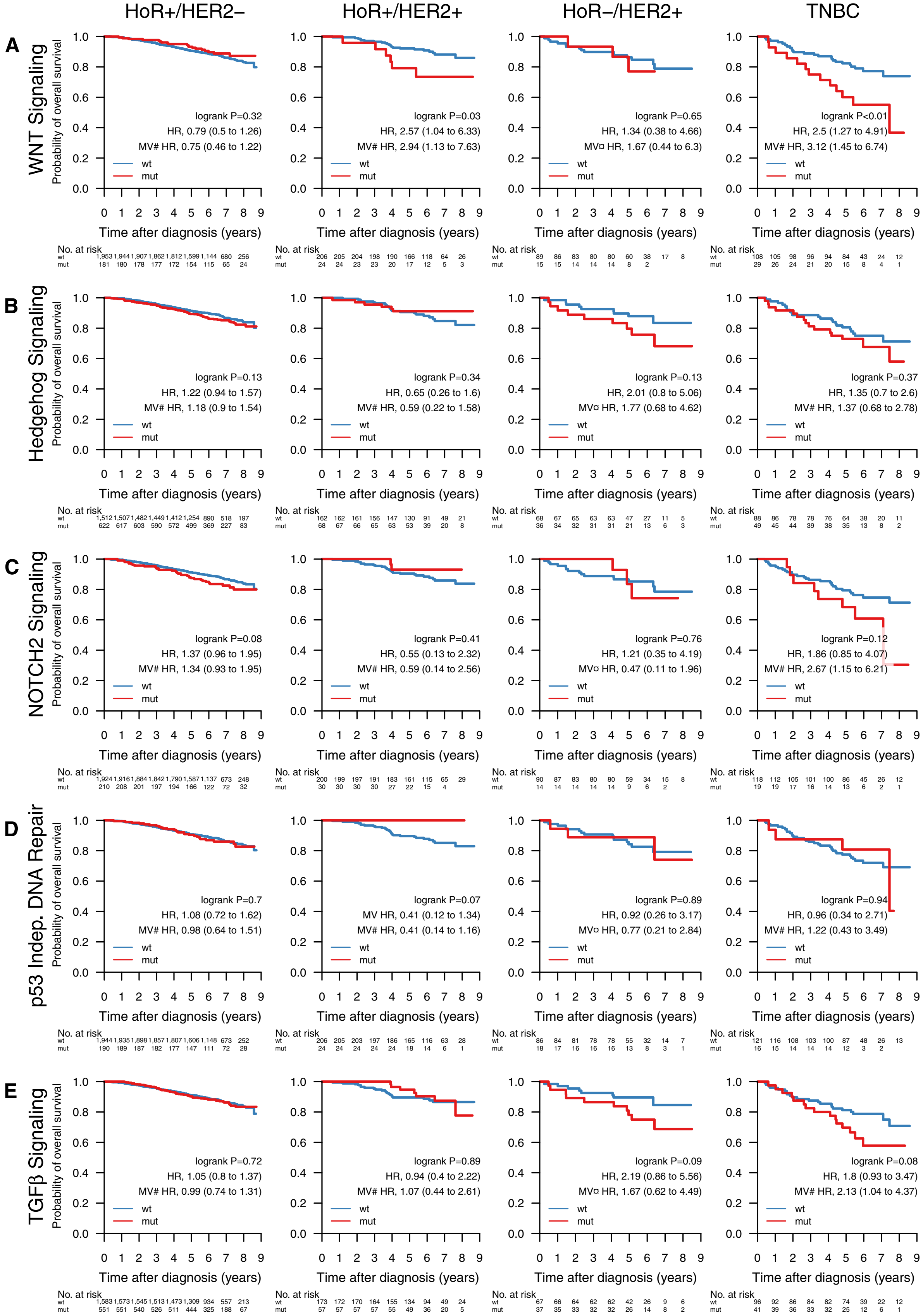
HoR+/HER2−
HoR+/HER2+
HoR−/HER2+
TNBC
A
WNT Signaling
B
Hedgehog Signaling
C
NOTCH2 Signaling
D
p53 Indep. DNA Repair
E
TGFβ Signaling
Probability of overall survival
Time after diagnosis (years)
No. at risk
wt
mut
logrank P=0.32
HR, 0.79 (0.5 to 1.26)
MV# HR, 0.75 (0.46 to 1.22)
logrank P=0.03
HR, 2.57 (1.04 to 6.33)
MV# HR, 2.94 (1.13 to 7.63)
logrank P=0.65
HR, 1.34 (0.38 to 4.66)
MV¤ HR, 1.67 (0.44 to 6.3)
logrank P<0.01
HR, 2.5 (1.27 to 4.91)
MV# HR, 3.12 (1.45 to 6.74)
logrank P=0.13
HR, 1.22 (0.94 to 1.57)
MV# HR, 1.18 (0.9 to 1.54)
logrank P=0.34
HR, 0.65 (0.26 to 1.6)
MV# HR, 0.59 (0.22 to 1.58)
logrank P=0.13
HR, 2.01 (0.8 to 5.06)
MV¤ HR, 1.77 (0.68 to 4.62)
logrank P=0.37
HR, 1.35 (0.7 to 2.6)
MV# HR, 1.37 (0.68 to 2.78)
logrank P=0.08
HR, 1.37 (0.96 to 1.95)
MV# HR, 1.34 (0.93 to 1.95)
logrank P=0.41
HR, 0.55 (0.13 to 2.32)
MV# HR, 0.59 (0.14 to 2.56)
logrank P=0.76
HR, 1.21 (0.35 to 4.19)
MV¤ HR, 0.47 (0.11 to 1.96)
logrank P=0.12
HR, 1.86 (0.85 to 4.07)
MV# HR, 2.67 (1.15 to 6.21)
logrank P=0.7
HR, 1.08 (0.72 to 1.62)
MV# HR, 0.98 (0.64 to 1.51)
logrank P=0.07
MV HR, 0.41 (0.12 to 1.34)
MV# HR, 0.41 (0.14 to 1.16)
logrank P=0.89
HR, 0.92 (0.26 to 3.17)
MV¤ HR, 0.77 (0.21 to 2.84)
logrank P=0.94
HR, 0.96 (0.34 to 2.71)
MV# HR, 1.22 (0.43 to 3.49)
logrank P=0.72
HR, 1.05 (0.8 to 1.37)
MV# HR, 0.99 (0.74 to 1.31)
logrank P=0.89
HR, 0.94 (0.4 to 2.22)
MV# HR, 1.07 (0.44 to 2.61)
logrank P=0.09
HR, 2.19 (0.86 to 5.56)
MV¤ HR, 1.67 (0.62 to 4.49)
logrank P=0.08
HR, 1.8 (0.93 to 3.47)
MV# HR, 2.13 (1.04 to 4.37)

**Figure EV3.**

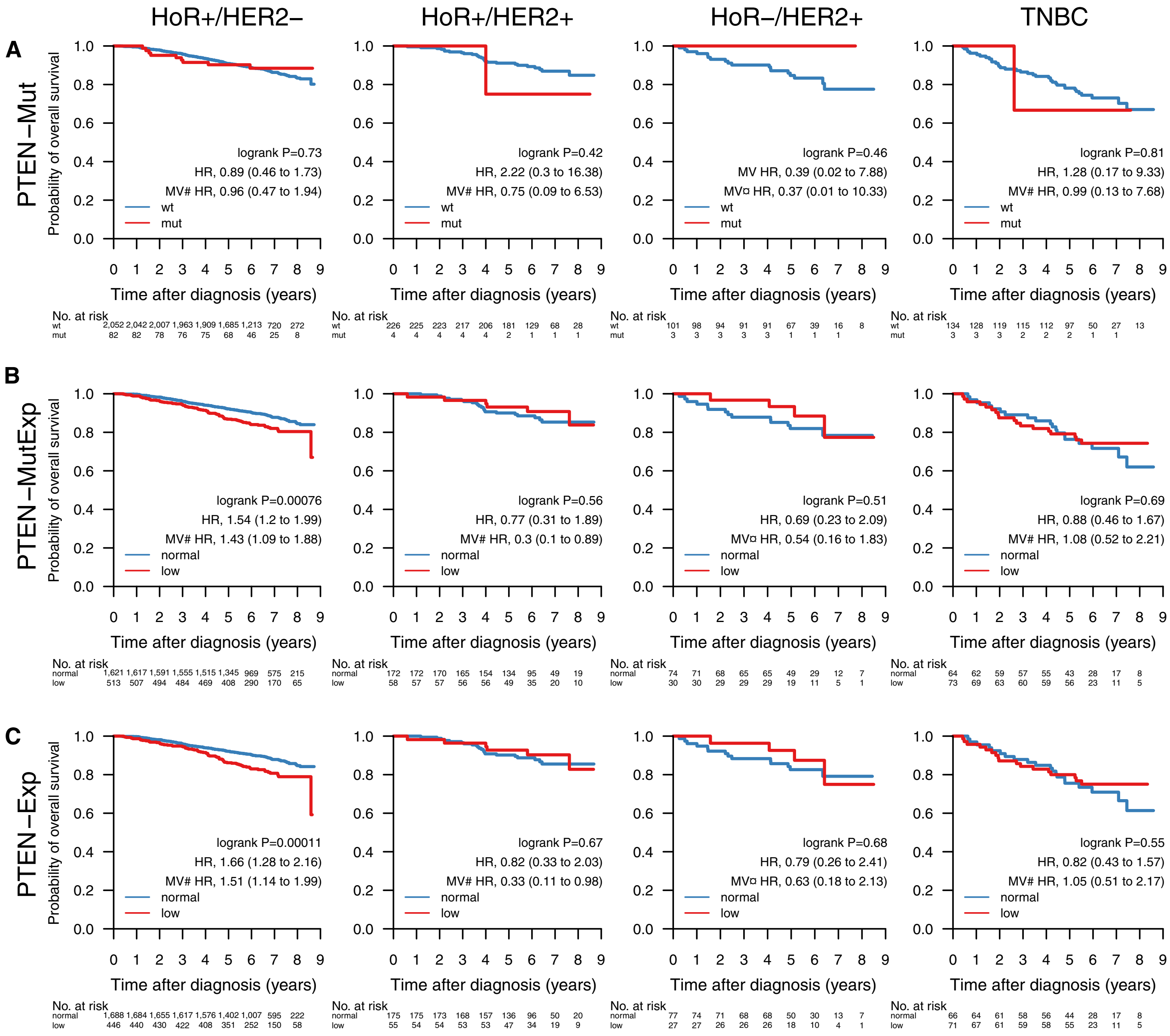


**Figure EV4. Impact of *PTEN* mutation and expression on overall survival across treatment groups.**

A–C Overall survival of patients with tumors containing a (A) *PTEN* mutation (*PTEN*-Mut), (B) a *PTEN* mutation and/or *PTEN* expression in the lower cohort-quartile (*PTEN*-MutExp), or (C) *PTEN* expression in the lower cohort-quartile (*PTEN*-Exp) in the clinical patient subgroups $HoR^{+}/HER2^{-}$ ($HoR^{+}$ when $ER^{+}$ and $PgR^{+}$, $HoR^{-}$ otherwise; *n* = 2,134), $HoR^{+}/HER2^{+}$ (*n* = 230), $HoR^{-}/HER2^{+}$ (*n* = 104), and TNBC (*n* = 137). Specific treatments in these groups are detailed in Table EV4. In each Kaplan–Meier plot, wild-type (wt) and normal cases are plotted in blue, mutated (mut) and low cases are plotted in red, the log-rank *P* value is given, and the hazard ratio (HR) for mutation/low is given with a 95% CI and after univariable and multivariable (MV) Cox regression adjustment. Covariables included in the MV analysis were age at diagnosis, lymph node status, tumor size, and the variables denoted by the following symbols: ¤, ER, PgR, and NHG; #, NHG. ER, estrogen receptor; HoR, hormone receptor; HER2, human epidermal growth factor receptor 2; NHG, Nottingham histological grade; PgR, progesterone receptor; TNBC, triple-negative breast cancer.


© The Authors

**Figure EV5. Impact of tumor mutational burden on overall survival across clinical subgroups.**

Association of tumor mutational burden (TMB) with overall survival in 3,217 patients, and within the biomarker patient subgroups $ER^+$, $ER^-$, $PgR^+$, $PgR^-$, HER2 amplified, HER2 normal, Ki67-high, Ki67-low, NHG Grade 1–3, $HoR^+/HER2^-$, $HoR^+/HER2^+$, $HoR^-/HER2^+$, TNBC, and molecular subtypes Luminal A and B, HER2-enriched, and basal-like according to the PAM50 gene list. In each Kaplan–Meier plot, TMB-low cases are plotted in blue, TMB-high cases are plotted in red, the log-rank *P* value is given, and the hazard ratio (HR) for TMB-high is given with a 95% CI and after univariable and multivariable (MV) Cox regression adjustment. Covariables included in the MV analysis were age at diagnosis, lymph node status, tumor size, and the variables denoted by the following symbols: ¶, ER, PgR, HER2, and NHG; ^, ER, PgR, HER2; ¤, ER, PgR, and NHG; $, HER2 and NHG; #, NHG. ER, estrogen receptor; HER2, human epidermal growth factor receptor 2; HoR, hormone receptor; NHG, Nottingham histological grade; PgR, progesterone receptor; TMB, tumor mutational burden; TNBC, triple-negative breast cancer.

© The Authors

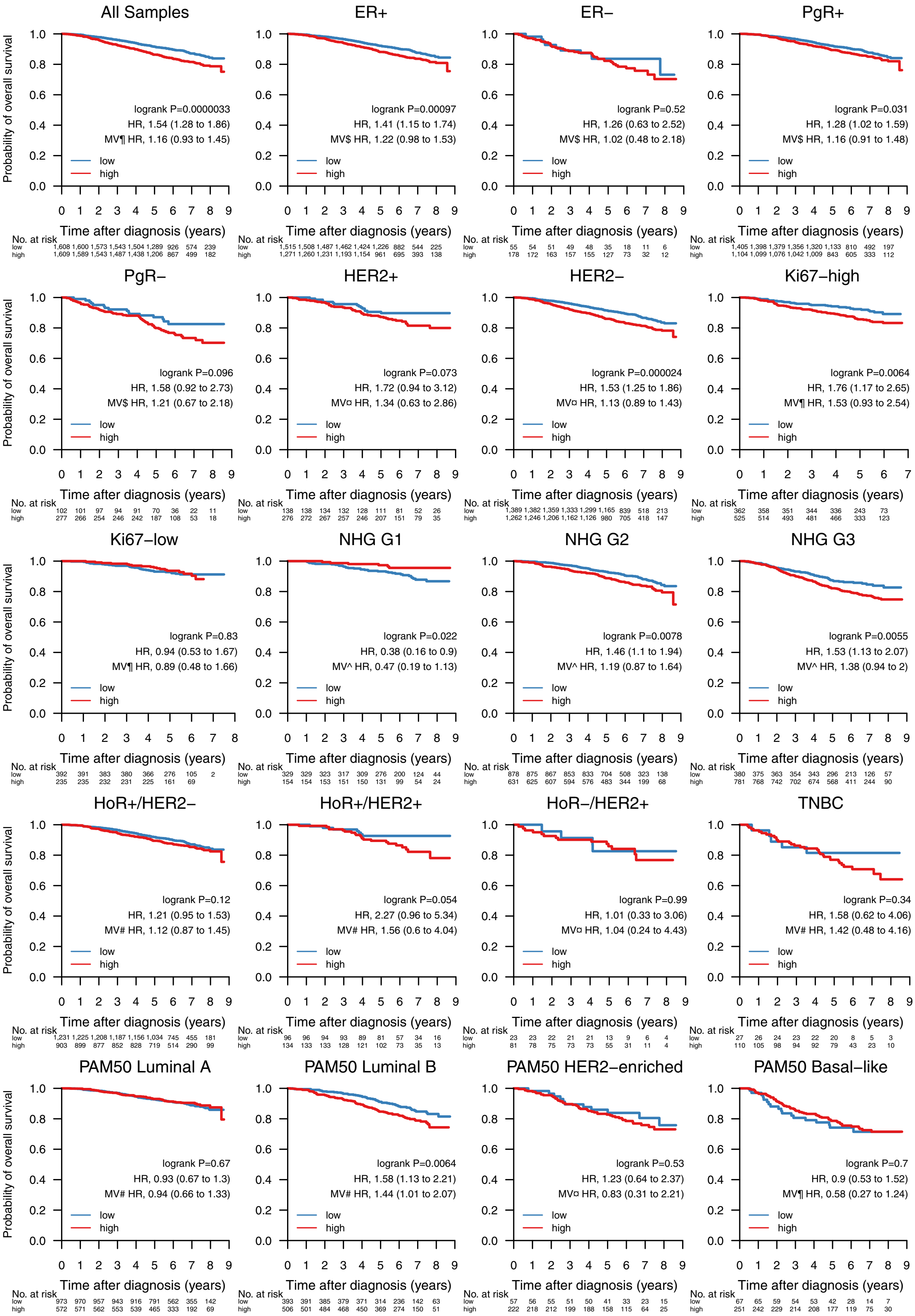


**Figure EV5.**



© The Authors
